# miR290-5p/292-5p Activate the Immunoglobulin *kappa* Locus in B Cell Development

**DOI:** 10.1371/journal.pone.0043805

**Published:** 2012-08-23

**Authors:** Patty B. Garcia, Amie Cai, Jamie G. Bates, Hector Nolla, Mark S. Schlissel

**Affiliations:** 1 Department of Molecular and Cell Biology, University of California, Berkeley, California, United States of America; 2 Department of Biochemistry, Stanford University School of Medicine, Stanford, California, United States of America; National Institute on Aging, United States of America

## Abstract

Regulated expression of miRNAs influences development in a wide variety of contexts. We report here that miR290-5p (100049710) and miR292-5p (100049711) are induced at the pre-B stage of murine B cell development and that they influence assembly of the Igκ light chain gene (243469) by contributing to the activation of germline Igκ transcription (κGT). We found that upon forced over-expression of miR290-5p/292-5p in Abelson Murine Leukemia Virus (AMuLV) transformed pro-B cells, two known activators of κGT, E2A (21423) and NF-κB (19697), show increased chromosomal binding to the *kappa* intronic enhancer. Conversely, knockdown of miR290-5p/292-5p in AMuLV pro-B cells blunts drug-induced activation of κGT. Furthermore, miR290-5p/292-5p knockdown also diminishes κGT activation, but not Rag1/2 (19373, 19374) expression, in an IL-7 dependent primary pro-B cell culture system. In addition, we identified a deficiency in κGT induction in miR290 cluster knockout mice. We hypothesize that increased expression of miR290-5p and miR292-5p contributes to the induction of κGT at the pre-B stage of B cell development through increased binding of NF-κB and E2A to *kappa* locus regulatory sequences.

## Introduction

Recent work implicates microRNAs (miRNAs) in the regulation of B cell development [1] [2], [3]. miRNAs are small non-coding RNAs, approximately 20–25 nucleotides in length processed from longer precursors, that exert sequence-targeted post-transcriptional repression of target transcripts [4], [5]. Primary miRNA transcripts are processed in the nucleus by an RNaseIII enzyme Drosha (14000), then exported to the cytoplasm for further processing by a second such enzyme, Dicer [4] (192119). Dicer selects a mature ∼22 nt miRNA strand that serves as effector in the RNA-induced silencing complex (RISC) to regulate target transcripts. Mice with B cell lineage-specific deletion of Dicer exhibit a developmental block at the pro-B stage of development [1]. This finding implicates the miRNA pathway and its effector members as playing an essential role at this stage and highlight the important function of miRNAs at the pro-B to pre-B transition, a critical checkpoint in B cell development. Although some miRNAs and their functions have been described [2], [3] further studies are needed to thoroughly identify miRNAs regulating B cell development.

miR290-5p and miR292-5p are members of the miR290 polycistronic cluster [6]. The miR290 cluster is expressed as a single transcript encoding seven miRNAs. miR290-5p/292-5p share the seed sequence CUCAAA similar to miR291-5p (100049715, 100124471), AUCAAA. This indicates that they are similar in function. The remaining miR290 cluster members that share the seed sequence AAGUCC are expressed under different contexts. These miRNAs are robustly expressed together in eutherian embryonic stem cells and have therefore been called the Early Embryonic microRNA Cluster (EEmiRC) [6]. Generally the CUCAAA miR290 cluster members and the AAGUCC members are not thought to overlap functionally.

The miR290 cluster germline knockout displays partially penetrant embryonic lethality in which homozygotes survive gestation at only 7% of the predicted Mendelian ratio [7]. Medeiros et al. hypothesize that the phenotype is partially penetrant in part due to the mixed background in their studies (129/C57BL6). Additionally, they point out that other miRNA deletions result in partially penetrant phenotypes, possibly due to random fluctuations of gene expression levels in the absence of the miRNAs. They further speculate that this is the case in the miR290 cluster deletion. A role for miR290 cluster members in lymphoid cells has not been described.

Antibody-secreting B cells are an essential component of the adaptive immune response [8]. The genes that encode antibody heavy- and light-chains are generated during B cell development through a complex and highly regulated process called V(D)J Recombination [9]. One of the key checkpoints during this process is the pro-B to pre-B transition. The immunoglobulin heavy chain (IgHC) locus (111507) must rearrange to encode a functional heavy chain protein for a pro-B cell to progress to the pre-B stage [10]. Once a functional IgHC protein is produced and successfully transits to the surface, early pre-B cells undergo a burst of proliferative expansion before exiting the cell cycle and commencing to rearrange the light chain immunoglobulin loci, *kappa* or *lambda* [11], [12] (111519). Transcription occurs across the unrearranged *kappa* locus prior to rearrangement generating what are known as germline *kappa* transcripts (κGT) [13]. The appearance of these transcripts indicates a *kappa* locus that is in an open chromatin state, available for access by the V(D)J recombinase proteins, Rag1 and Rag2 [14]. *Kappa* locus activation is tightly regulated during B cell development.

The activation and rearrangement of the *kappa* locus requires two *kappa* locus enhancers, the kappa intronic enhancer (Eκi) and the 3′ kappa enhancer (3′Eκ) [15]. Transcription factors that enhance *kappa* locus activation through binding to these enhancers include E2A, which binds to both enhancers [16], NF-κB, which binds Eκi [17], [18], and Pax5 (18507), which binds 3′Eκ and has been shown to be necessary for *kappa* rearrangements [19]. Binding of these transcription factors to the *kappa* locus enhancers is regulated as cells develop in the bone marrow. Signaling through the IL-7 receptor and pre-B Cell Receptor (pre-BCR) concomitantly direct transcription factors to bind either enhancer, thereby activating κGT and *kappa* rearrangements [20], [21]. While much is known about signaling through these pathways, nothing is known about the role miRNAs play in *kappa* locus activation at the pro-B to pre-B transition.

We sought to identify miRNAs that play a role in the pro-B to pre-B transition using AMuLV-transformed pro-B cells as a model system. AMuLV transformation immortalizes developing B cells at the pro-B to large pre-B stages of B cell development [22], [23]. The immortalized cells are blocked in differentiation and receive survival signals from constitutive v-Abl [24] kinase activity, independent of IL7 (16196) cytokine signals [25]. Studies by our lab and others have shown that inactivation of the constitutive Abl kinase activity, by the small molecule Abl-kinase inhibitor STI571 (also known as Gleevec), in AMuLV cell lines results in G1 cell cycle arrest, progression to a pre-B-like stage, and eventual apoptosis [25]. The transcripts induced upon STI571 treatment include pre-B stage-specific genes such as SpiB (272382), IRF4 (16364), Rag1/2, and κGT, confirming that Abl kinase activity blocks B cell differentiation. While pre-B stage-specific genes have been identified in this system, miRNAs specific to this stage have yet to be described.

In this study, we screened for miRNAs that are upregulated in STI571-treated AMuLV-transformed pro-B cells and that promote B cell differentiation. Our screen identified two miRNAs, miR290-5p and miR292-5p, that originate from the miR290 Cluster transcript and share an identical seed-sequence. miR290-5p and miR292-5p are independently able to induce expression of κGT expression as well as increase DNA binding activity of NF-κB and E2A. These transcription factors bind directly to their cognate sites in the intronic *kappa* enhancer. We propose a novel role for miR290-5p and miR292-5p in the activation of the *kappa* locus during B cell development.

## Results

### microRNA Screen in AMuLV proB Cells

To identify miRNAs that may be of importance at the pro-to-pre-B cell transition of B cell development we analyzed RNA purified from AMuLV-transformed pro-B cell lines. When AMuLV-transformed pro-B cells are treated with STI571, they progress to a pre-B-like state of development [25]. To broadly identify miRNAs that change in expression levels upon the addition of STI571, we used the Exiqon microarray platform to screen for such miRNA populations in three independent AMuLV transformants (E2A+/+, 220−8, 63−12). We cultured each line in the presence or absence of STI571 for 12 hrs (2.5 µM), purified total RNA from these samples, and subjected them to microarray analysis (GSE38331).

**Figure 1:**
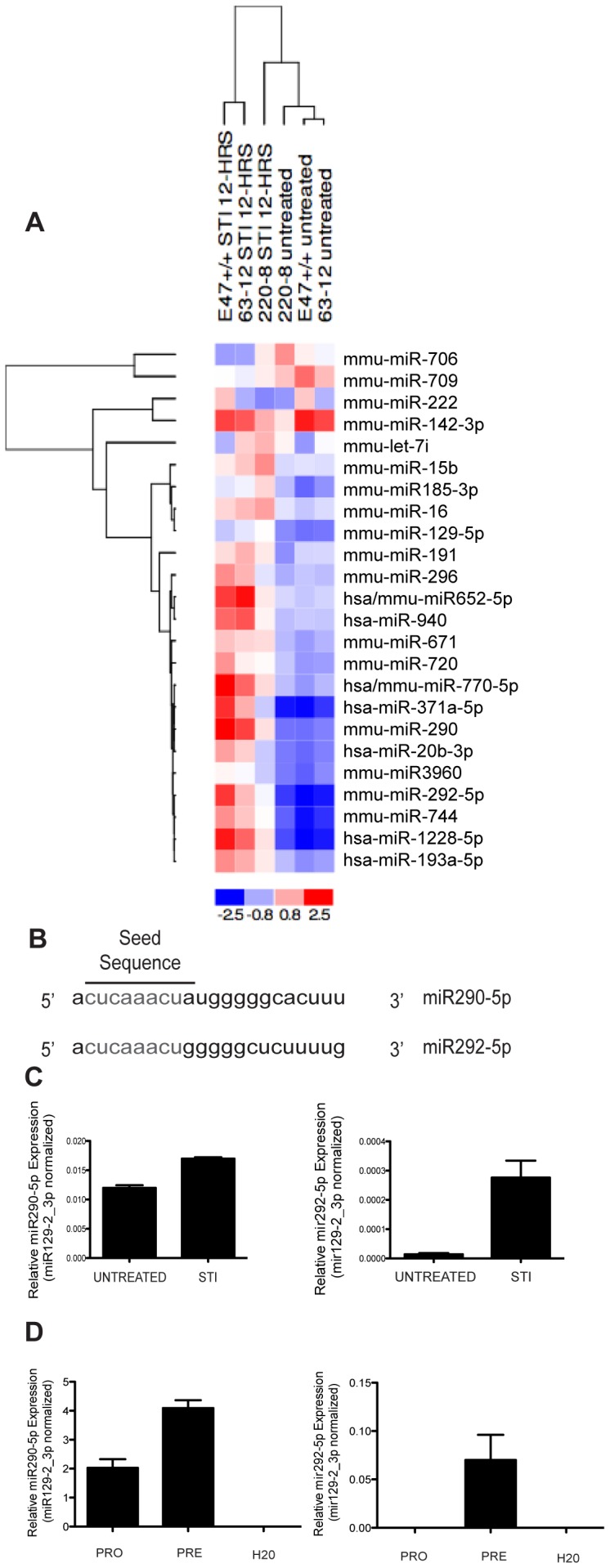
miR290-5p and miR292-5p are induced at the pro-B to pre-B transition. 1A. Heat-map representing levels of miRNAs from a microarray analysis of RNA purified from the indicated AMuLV cell lines cultured in the absence or presence of STI571 (2.5 µM, 12 hr). Performed once in three independently transformed cell lines. 1B. Schematic depiction of the shared seed sequence of the mature miR290-5p and miR292-5p microRNAs. 1C. qPCR analysis of miR290-5p or miR292-5p expression levels in RNA purified from E2A+/+ AMuLV cells cultured in the absence or presence of STI571 (2.5 µM, 12 hr). Data was normalized to the expression of miR129-2_3p. Error bars represent range for replicate qPCR reactions. The data shown is from one representative experiment of three biological replicates. 1D. qPCR analysis of miR290-5p or miR292-5p in primary wild-type pro-B (B220+, CD43+, IgM−) cells or pre-B (B220+, CD43−, IgM−) cells. Data was normalized to the expression of miR129-2_3p. Error bars represent range for replicate qPCR reactions. Data shown is from one representative experiment, of three independent sort experiments.

The general trend observed was an increase in expression of miRNAs upon STI571 treatment. However, there were a few miRNAs that decreased in expression upon STI571 treatment (Fig. 1a). Two of the miRNAs identified in the screen as having expression consistently and significantly increased across all three independently transformed AMuLV-transformed pro-B cell lines (Fig. 1a) were miR290-5p and miR292-5p. These miRNAs are members of the murine miR290 polycistronic cluster, previously reported to be expressed only in gonads of both sexes, but not in other tissues examined [7]. They share the same seed sequence (Fig. 1b.) indicating a possible functional redundancy within pre-B cells.

To validate the results of the microarray, we cultured E2A+/+ AMuLV cells in the presence or absence of STI571 for 12 hrs (2.5 µM). Using specific Taqman qPCR assays, we confirmed that both miR290-5p and miR292-5p display a modest increase in expression upon STI571 treatment (Fig. 1c).

We next asked whether these miRNAs displayed a similar pattern of regulated expression in wild-type primary developing pro- and pre-B cells. We sorted pro-B (B220+, CD43+, IgM−) and pre-B (B220+, CD43−, IgM−) cells from wild-type mouse bone marrow by flow cytometry, purified total RNA, and performed qPCR for quantification of miR290-5p and miR292-5p. We found that the increase in expression we observed in STI571 treated AMuLV-transformed pro-B cells, was mirrored in primary pro-B and pre-B cells (Fig. 1d). A third member of the miR290 polycistronic cluster with a similar seed sequence as miR290-5p/292-5p, miR291-5p, is also induced; however other members of the miR290 cluster with an alternate seed sequence did not increase upon STI571 treatment (Figure S1).

### Over-expression of miR290-5p or miR292-5p Induces Germline Igκ Transcription in AMuLV Cells

**Figure 2:**
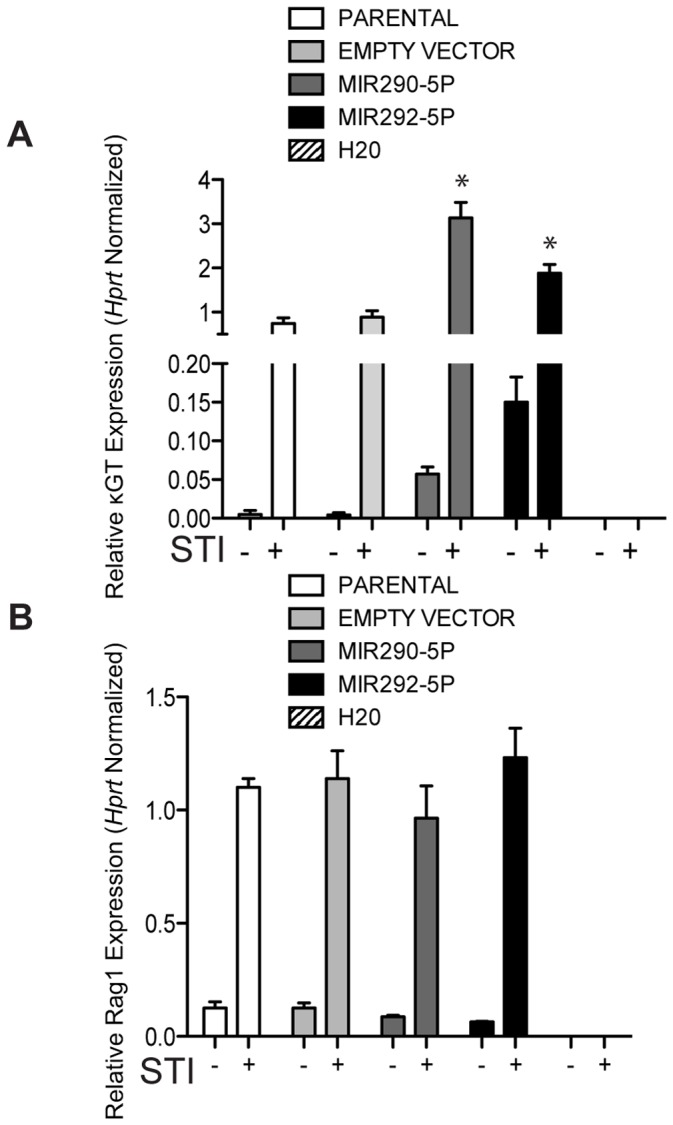
Over-expression of miR290-5p or miR292-5p induces κGT expression in AMuLV cells. 2A, B. qPCR analysis of (A) κGT or (B) Rag1 expression in RNA purified from E2A+/+ AMuLV cells over-expressing either miR290-5p or miR292-5p, cultured in the absence or presence of STI571 (2.5 µM, 12 hr). The (A) κGT qPCR y-axis reflects two different scales. Data was normalized to the expression of *Hprt*. Error bars represent range for replicate qPCR reactions. Asterisk represents P value <0.05. The P value was derived by the Student’s T test. Data shown is from one representative experiment of at least four individual experiments.

Previous studies have shown that the addition of STI571 to AMuLV-transformed pro-B cells induces expression of genes characteristic of the pre-B cell stage in development [25]. Since these miRNAs increase in expression upon STI571 treatment, we hypothesized that they may play a role in regulating developmental progression at the pre-B cell stage. In order to probe the role that these miRNAs might play at this drug-induced developmental transition, we cloned each miRNA in its genomic context into an MSCV-based IRES-CD2 marked retroviral expression vector. We transduced the E2A+/+ AMuLV cell line with each miRNA vector or an empty vector control and asked whether the miRNA-overexpressing cells activate transcription of the germline Igκ locus transcript, κGT. The activation of the Igκ locus is a hallmark of the pre-B stage of development that is tightly correlated with the activation of V-to-Jκ rearrangement [13]. Upon stable over-expression of either miR290-5p or mir292-5p, we observed an induction in κGT (Fig. 2a), but not to the levels seen in wild-type AMuLV-transformed pro-B cells treated with STI571. However, when we treated the miRNA-transduced cell lines with STI571 (12 hrs, 2.5 µM), we saw a super-induction of κGT (Fig. 2a). These data indicate that either miRNA is able to independently act upon a pathway that results in activation of the germline Igκ locus. In contrast, neither miRNA altered the induced or basal levels of *Rag1* transcription in these same cells (Fig. 2b).

### miRNA Knockdown Partially Blunts the Induction of κGT upon v-Abl Inhibition

To confirm that the apparent role of miR290-5p and miR292-5p in the activation of κGT observed in these experiments was not an artifact of over-expression, we generated miRNA sponge constructs to perform knockdown experiments. These sponge constructs were generated by independently cloning three imperfect miRNA binding sites for each miRNA into the 3′UTR of a GFP (7011691) cDNA [24]. We also cloned a sponge construct for a different miRNA, miR129-2_3p (723953), to use as a negative control. miR129-2_3p is expressed at similar levels as miR290-5p and miR292-5p at the pre-B stage, and is not induced at the pro-to pre-B stage in primary B cells or upon STI571 treatment of AMuLV-transformed pro-B cells (data not shown). We generated stable AMuLV-transformed cell lines transduced with retroviral vectors expressing these sponge constructs to examine their effects on κGT expression.

To first confirm that the sponge constructs were engaging with the target miRNA, we stably transduced AMuLV cells with GFP miRNA-sponge constructs marked with a dual tomato red cDNA. The expression of the tandem tomato red cDNA was independent of miRNA sponge regulation and serves for normalization against the variable GFP expression. Upon STI treatment (42 hrs, 1 µM) we observed a decrease in GFP expression relative to tomato red expression, in the miR290-5p or miR292-5p sponges, suggesting that the sponges were engaging with the target miRNAs (Figure S2).

**Figure 3:**
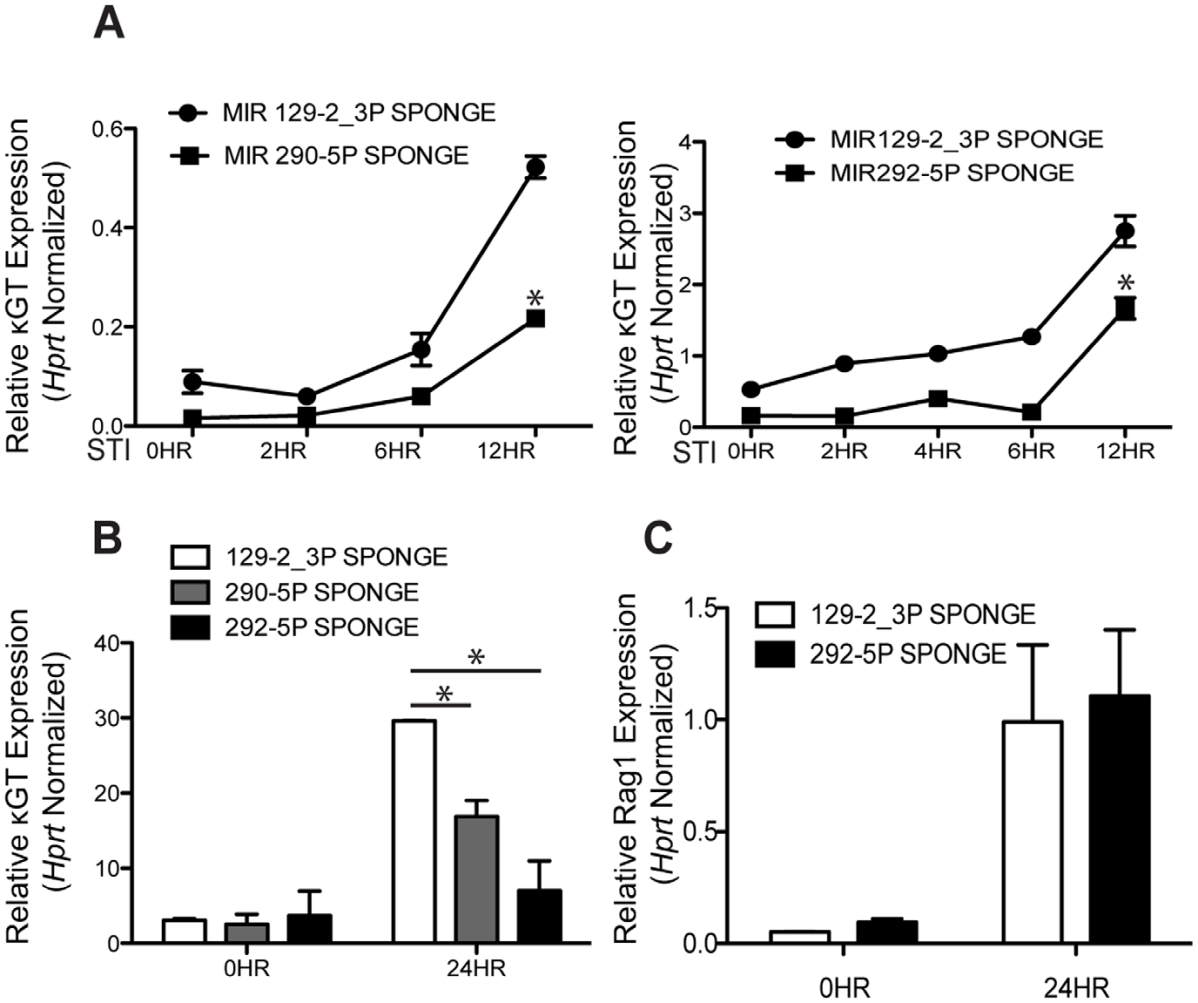
κGT induction is blunted upon knockdown of miR290-5p or miR292-5p. 3A. qPCR analysis of κGT expression in RNA purified from E2A+/+ AMuLV cells expressing a miR290-5p or miR292-5p sponge knockdown construct, and cultured in the presence of STI571 (2.5 µM) for the indicated lengths of time. Data was normalized to the expression of *Hprt*. Error bars represent range for replicate qPCR reactions. Asterisk represents P value <0.05. The P value was derived by the Student’s T test. These data are from one representative experiment of at least four independently performed experiments. 3B, C. qPCR analysis of (B) κGT or (C) Rag1 expression in RNA purified from wild-type primary pro-B cells transduced with either a miR129-2_3p control knockdown sponge, or a miR290-5p or miR292-5p knockdown sponge. Wild-type primary pro-B cells were cultured in rIL7 (2 ng/ml) for 5 days and then transduced with indicated sponge construct. Marker positive cells were then sorted and IL7 withdrawn from culture for 24 hrs before RNA was harvested. qPCR measures (B) κGT or (C) Rag1 expression in cells before or after the withdrawal of IL7. Data was normalized to *Hprt* expression levels. Error bars represent range for replicate qPCR reactions. Asterisk represents P value <0.05. The P value was derived by the Student’s T test. These data show one representative experiment of at least four independently performed experiments.

We then examined the sponge effects on κGT expression. We cultured the stable GFP sponge cell lines with STI571 (12 hrs, 2.5 µM) and observed a normal induction of κGT in the negative control sponge cell line (Fig. 3a). However, we observed a blunting of normal κGT induction in the miR290-5p or miR292-5p sponge lines (Fig. 3a). This blunting of κGT induction upon knockdown of endogenous drug-induced miR290-5p or miR292-5p indicates that these miRNAs partially contribute to the activation of the *kappa* locus upon STI571 treatment of AMuLV-transformed cells.

### miRNA Knockdown Partially Blunts κGT Induction in Primary Cell Culture

To further test the role of miR290-5p and miR292-5p in the activation of the *kappa* locus observed in our AMuLV model system, we performed similar sponge knockdown experiments in a primary developing B cell culture system. Cultured primary pro-B cells proliferate in the presence of high concentrations of rIL7 but do not differentiate, while at low concentrations of rIL7, the cells exit the cell cycle and progress to the pre-B cell stage of development [20]. We harvested bone marrow from wild-type mice and cultured cells in 2 ng/ml rIL7 for 5 days to expand the pro-B cell population. We then retrovirally transduced the expanded pro-B cell culture with either the negative control, miR290-5p, or miR292-5p sponges. After an additional 24 hours in rIL7 culture, we harvested a portion of each population as a “0 hour” time-point. We then washed the remaining cells and re-cultured them in the absence of rIL7 for 24 hr.

In cells transduced with the negative control sponge, there was normal induction of κGT upon IL-7 withdrawal as expected (Fig. 3b). However, in the presence of the miR290-5p or miR292-5p sponges, IL7 withdrawal resulted in a significantly diminished induction of κGT expression (Fig. 3b). We observed indistinguishable induction of *Rag1* gene expression upon IL7 withdrawals in miR292-5p as compared to miR129-2_3p negative control sponge cultures (Fig. 3c). Together these data indicate that miR290-5p and miR292-5p independently contribute to the induction of κGT expression in primary pre-B cells.

### miR290-5p and miR292-5p Enhance DNA Binding Activity of E2A and NF-κB

In an effort to elucidate the pathway through which miR290-5p or miR292-5p induce κGT, we examined transcription factors known to be involved in the induction of κGT by direct binding to either the 3′ *kappa* enhancer (3′Eκ) or the *kappa* intronic enhancer (Eκi). Among them, E2A and NF-κB bind Eκi while E2A can also bind 3′Eκ [16], [18,18]. To determine if miR290-5p/292-5p over-expression increases expression of E2A, we performed qPCR. We did not observe an increase in E2A mRNA levels upon miR290-5p/miR292-5p over-expression. (Figure S3).

Since both E2A and NF-κB binding activity can be regulated post-transcriptionally, we went on to ask whether miR290-5p/292-5p regulate the induction of κGT by inducing DNA binding activity of E2A and NF-κB to their target sequences in the *kappa* locus. To approach this we performed Chromatin Immunoprecipitation (ChIP).

**Figure 4:**
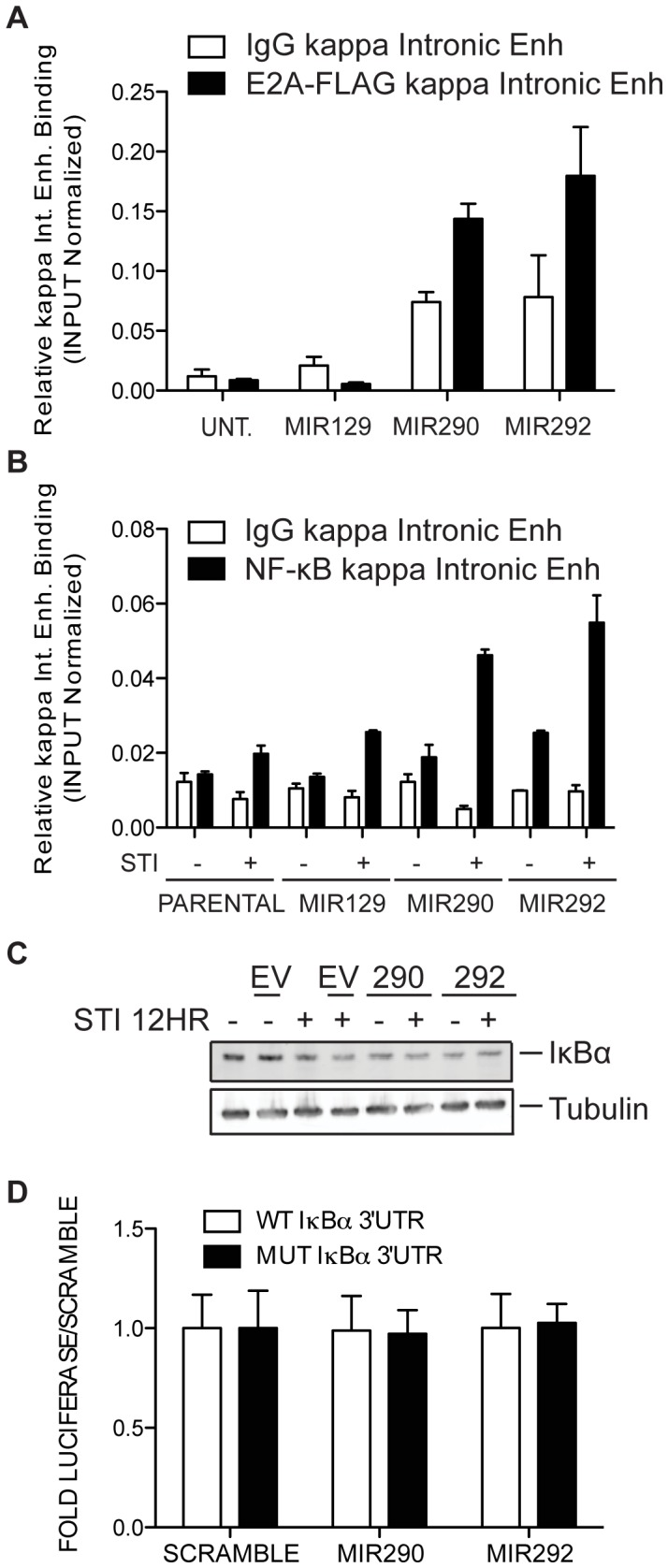
Over-expression of miR290-5p or miR292-5p induces activation of E2A and NF-κB. 4A. ChIP analysis of HF4 AMuLV cells expressing His-FLAG-E2A, at endogenous levels, and over-expressing either miR129-2_3p, miR290-5p, or mir292-5p. Chromatin samples were immunoprecipitated with anti-FLAG or IgG control antibody. Relative enrichment of bound DNA over input was determined by subjecting precipitates to qPCR with primers specific to the Eκi binding region for E2A. This data shows one representative experiment of two independent experiments. 4B. ChIP analysis of E2A+/+ AMuLV cells over-expressing either miR129-2_3p, miR290-5p, or mir292-5p, in the absence or presence of STI571 (1 µM, 16 hr). Chromatin samples were immunoprecipitated with anti-p50 or IgG control antibody. Relative enrichment of bound DNA over input was determined by subjecting precipitates to qPCR with primers specific to the Eκi binding region for NF-κB. This data shows one representative experiment of two independent experiments. 4C. Immunoblot analysis of E2A+/+ AMuLV cells expressing either an empty vector control, miR290-5p, or miR292-5p and cultured in either the absence or presence of STI571 (2.5 µM, 12 hr). Immunoblot was probed with anti-IκBα and anti-Tubulin antibodies. 4D. Luciferase assay of total cell lysates from HEK293 cells transiently transfected with either a wild-type IκBα 3′UTR reporter or a mutant IκBα 3′UTR reporter along with a scrambled miRNA, mir290-5p or miR292-5p. Error bars represent range for biological replicate luciferase reactions. Data shows one representative experiment of at least three independent experiments.

It was reported previously that upon IL7 withdrawal, the amount of bound E2A increases at Eκi [20] and knockdown of miR290-5p or mir292-5p upon IL7 withdrawal blunts κGT induction as shown above. So we asked if miR290-5p or miR292-5p over-expression enhances E2A binding to Eκi in vivo. We made use of the HF4 AMuLV-transformed pro-B cell line generated from a mouse that expresses a FLAG-tagged E2A knocked into the E2A locus [26], [27]. We stably expressed miR129-2_3p, miR290-5p, or miR292-5p in this cell line and performed ChIP with anti-FLAG or anti-IgG antibodies. Using qPCR to analyze the precipitated DNA, we observed increased E2A-FLAG binding to Eκi in both miR290-5p and miR292-5p over-expressing cells (Fig. 4a). These data indicate that miR290-5p or miR292-5p expression induces binding of E2A to Eκi within the *kappa* locus.

The gene encoding a known inhibitor of E2A activity, ID2 [28] (15902), has predicted binding sites for miR290-5p/miR292-5p in its 3′UTR. We sought to determine if miR290-5p/292-5p directly repress the ID2 3′UTR using a dual-luciferase assay. A luciferase mRNA fused to the ID2 3′UTR was not directly repressed by these miRNAs (Figure S4).

NF-κB, like E2A, binds Eκi [18]. We went on to examine whether NF-κB binding activity increases when these two miRNAs are over-expressed in E2A+/+ AMuLV lines cultured with STI (1 µM, 16 hr). We stably expressed miR129-2-_3p, miR290-5p, or miR292-5p in this cell line and performed ChIP with anti-p50 (18033) (an NF-κB subunit) or anti-IgG antibodies. We observed increased p50 binding to Eκi in both miR290-5p and miR292-5p over-expressing cells and this binding was further increased in the presence of STI571 (Fig. 4b). These data indicate that miR290-5p or miR292-5p expression induces binding of NF-κB to Eκi within the *kappa* locus.

To determine if factors upstream of NF-κB are affected by miR290-5p/miR292-5p expression, we examined IκBα (18035), a known inhibitor of NF-κB [29]. Protein levels for IκBα are repressed upon miR290-5p or miR292-5p over-expression to similar levels as observed in the STI571-treated control sample. This indicates that members of this pathway may be regulated by miR290-5p/292-5p (Fig. 4c).

We used a dual-luciferase assay to determine whether miR290-5p/292-5p can directly repress the IκBα 3′UTR. Luciferase activity was not repressed in cells overexpressing miR290-5p/292-5p by fusion of its cDNA to the IκBα 3′UTR (Fig. 4d).

### The miR290 Cluster in B Cell Development

**Figure 5:**
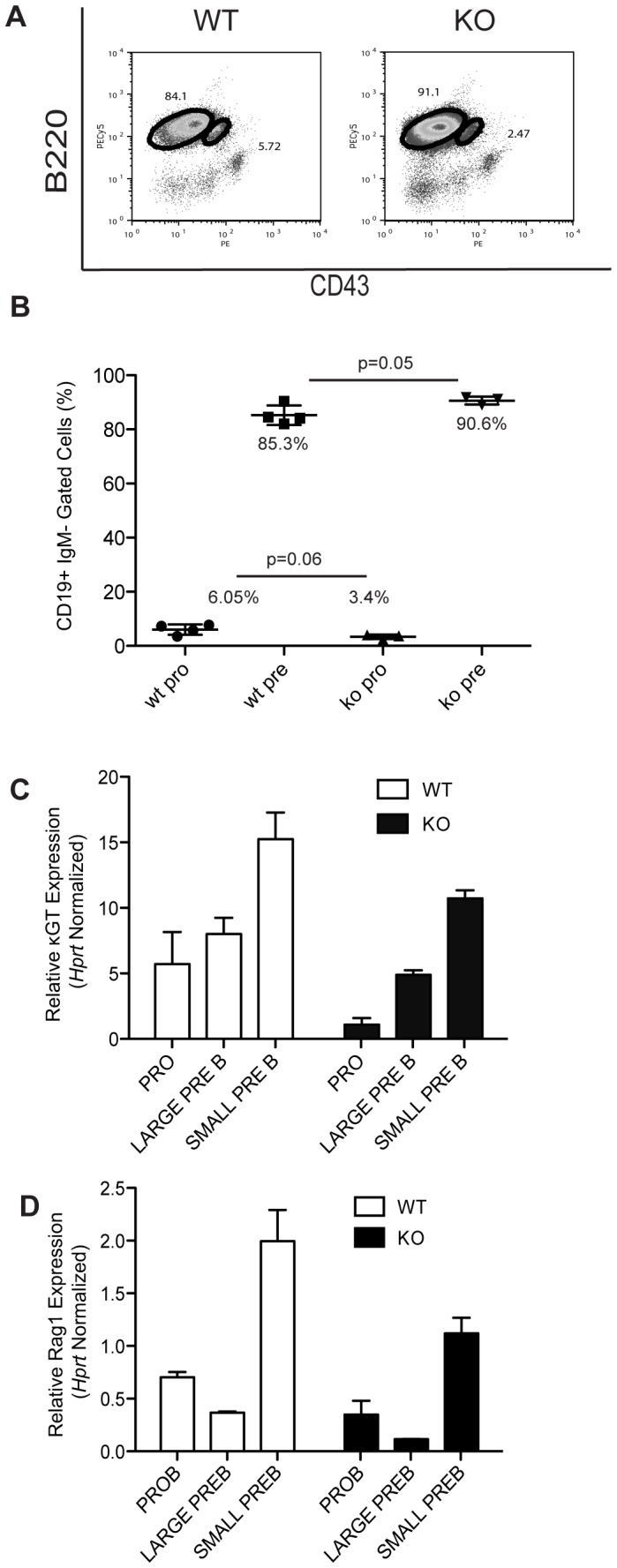
Germline knockout of the miR290 cluster affects the pre-B cell population. 5A. FACS analysis of 6 week old miR290 cluster knockout or wild-type mice. FACS plot reflects CD19 enriched cells gated on IgM negative cells. B220+, CD43+ correspond to pro-B cells and B220+, CD43− correspond to pre-B cells.; WT: pro-B, 5.72%, pre-B 84.1%; KO: pro-B, 2.47%, pre-B 91.1%. This experiment represents one of four independent experiments. Each experiment was with at least one mouse per genotype. 5B. Plot of the percentages of CD19+, IgM− pro-B (B220+, CD43+) and pre-B (B220+, CD43−) for each independent knockout and wild-type mouse analyzed as in Figure 5A. Average percentages are WT: pro-B, 6.05%, pre-B 85.3%; KO: pro-B, 3.4%, pre-B 90.6%. Line represents the average percentage for each population and the P value was derived by the Student’s T test. The average was derived from at least four mice per genotype. 5C, D qPCR analysis of (C) κGT or (D) Rag1 levels in RNA purified from flow-sorted pro-B (B220+, CD43+, IgM−), large pre-B (B220+, CD43−, IgM−, FSC-Hi), and small pre-B (B220+, CD43−, IgM−, FSC-Lo) cells. Mice were 6 week old miR290 cluster knockout or wild-type mice. The data was normalized to *Hprt* expression. Error bars represent range for replicate qPCR reactions. Here we show qPCR data from a representative mouse from each genotype. This experiment has been repeated with four mice per genotype and data shown is representative of three out of four miR290 knockout mice.

To confirm whether miR290-5p, miR292-5p, and more broadly the miR290 cluster are involved in B cell development, we analyzed B cell development in miR290 cluster knockout as compared to wild-type mice [7]. We analyzed total bone marrow by flow cytometry focusing on pro-B (B220+, CD43+, IgM−) (19264, 20737, 16019) and pre-B (B220+, CD43−, IgM−) populations. We found a moderate increase in the percentage of pre-B cells in the homozygous miR290 knockout mice, as compared to the wild-type mice (p = 0.05) (Fig. 5a, b).

To confirm our miRNA knockdown results that implicated both miR290-5p and miR292-5p as playing a role in activating the *kappa* locus, we used the miR290 cluster knockout mice to analyze κGT expression in pro-B, large pre-B (B220+, CD43−, IgM− FSC−Hi), and small pre-B cell (B220+, CD43−, IgM− FSC-Lo) populations. We observed a blunted κGT induction in the small pre-B population of miR290 cluster knockout mice (Fig. 5c). Interestingly, we also observed a blunted *Rag1* induction in the small pre-B population of miR290 cluster knockout mice (Fig. 5d). This was unexpected since we did not observe an effect on Rag1 in the pro-B culture experiments. This difference might be attributable to the other miR290 cluster members also deleted in the miR290 knockout.

Together, these data indicate that members of the miR290 polycistronic cluster, miR290-5p and miR292-5p are involved in the activation of the *kappa* locus as indicated by expression of κGT.

## Discussion

This study demonstrates that developing B cells induce expression of miR290-5p and miR292-5p at the pre-B stage to fully activate the germline Igκ locus, a critical event in the pro-to-pre-B cell transition. A modest induction of miR290-5p/292-5p occurs in response to STI571-inhibition of v-abl in transformed pro-B cell cultures, as well as in response to IL7 attenuation in primary B cell cultures. It is possible that miR290-5p/miR292-5p induction upon STI571-treatment of Abelson cells and IL7 attenuation in primary B cell cultures, is a consequence of either cell cycle arrest or activation of the apoptosis pathway known to occur under these circumstances. We reported previously, however, that activation of κGT and Rag1 transcription in STI571-treated Abelson cells is independent of both processes [25]. In addition, preliminary experiments show that miR290-5p/292-5p are induced at an earlier time point than the onset of cell cycle arrest in STI-571 treated Abelson cells, therefore, making it unlikely that this miRNA cluster is induced as consequence of cell cycle arrest.

The modest miR290-5p/292-5p induction may be a reflection of the partial role these miRNAs play in full κGT activation. NF-κB and E2A showed increased binding to Eκi *in vivo*, in response to miR290-5p/292-5p expression. Using over-expression, we found that miR290-5p/292-5p contribute to full activation of κGT. Knockdown strategies showed that miR290-5p/292-5p are necessary for full activation of κGT. In miR290 cluster knockout mice we observe diminished κGT levels in both pro-B and pre-B cells. Additional pre-B stage signals, such as other miRNAs, may be required to induce full expression of κGT [4]. v-abl inhibition or IL7 attenuation likely induce additional signaling pathways, aside from miRNAs, that are synergistic with miR290-5p/292-5p, and necessary for full κGT induction.

Previous work has shown that κGT are correlated with the activation of Igκ LC rearrangement [13], and may be required for progression from pre-B to immature B cells. We observed a deficiency in κGT induction in knockdown experiments from AMuLV-transformed pro-B cells and primary B cell cultures. In the miR290 cluster knockout mice we observed a modest accumulation of pre-B cells and a general blunting of κGT levels. Together, these observations support our conclusion that members of the miR290 polycistronic cluster play a role in the developmental activation of kGT expression.

### A miRNA-regulated Pathway for the Activation of the Igκ*appa* Locus

Previous work showed that IL7 attenuation allows for both expression of Rag1/2 as well as induction of κGT through E2A binding to Eκi [20]. Interestingly, we did not observe an increase in E2A mRNA levels upon miR290-5p/292-5p over-expression. We propose that E2A activation, upon IL7 attenuation, is regulated through miR290-5p/292-5p expression. We note that miR290-5p/292-5p does not regulate Rag1/2 expression in cell culture systems. Rag1 induction remained intact in both the AMuLV pro-B cells as well as the IL7-dependent primary B cell cultures, upon knockdown of miR290-5p or mir292-5p, while κGT induction was blunted. In miR290-5p/292-5p over-expressing cell lines we did not observe *kappa* locus rearrangements, despite κGT induction (data not shown). This may be explained by the lack of Rag1/2 activation when miR290-5p/292-5p are over-expressed in AMuLV pro-B cells in the absence of STI571. However, in the miR290 cluster knockout mice we observe a blunting of Rag1 induction at the pro-B, large pre-B, and small pre-B stages. This different effect on Rag1 may be attributed to the other miRNA members of the miR290 cluster also deleted in the miR290 cluster knockout mouse. However, aside from miR291-5p, we could not detect their expression in sorted pre-B cells of wild-type mice. Therefore, we propose that loss of IL7 signaling activates miR290-5p/292-5p, that leads to induction of κGT.

NF-κB, has been shown, by our group and others, to be highly expressed at the pre-B stage [30], [31]. We reported a correlation between the expression of both NF-κB and its inhibitor, IκBα, with κGT and light chain gene rearrangements. Furthermore, we reported that RAG1/2 expression is independent from NF-κB expression in pre-B cells. Activation of NF-κB is due in part to miR290-5p/292-5p induction at the pre-B stage.

The NF-κB inhibitor, IκBα [29], is an interesting candidate for direct targeting by miR290-5p/292-5p. In our studies we observed a decrease in IκBα protein expression upon miR290-5p/292-5p over-expression. Despite having predicted miR290-5p/miR292-5p binding sites in its 3′UTR, a luciferase mRNA fused to the IκBα 3′UTR was not directly repressed by these miRNAs. It is important to note that IκBα has a predicted miR290-5p/292-5p binding site in exon 4 that has not been examined. Likewise, a known inhibitor of E2A, ID2 [28], has predicted binding sites for miR290-5p/miR292-5p in its 3′UTR, but the ID2 3′UTR was not repressed by the miRNAs. It is possible that the target of miR290-5p/miR292-5p is an unknown inhibitor of one or both of these transcription factors, or a factor that is further upstream in the pathway. Additional studies are necessary to identify the relevant direct targets of these miRNAs in pre-B cells.

### miRNAs in B Cell Development

In this study, we show a role for miRNAs at the pre-B stage in B cell development. Several groups have shown that miRNAs are essential for the pro-to-pre-B transition, supporting our postulate that miRNAs regulate different stages of B cell development. Notably, Koralov [1] and colleagues deleted Dicer, an essential miRNA processing enzyme, in AMuLV-transformed pro-B cells and identified a set of upregulated transcripts. One such example, Bim (12125), a pro-apoptotic gene, was identified as a target of miR17-92 (723905) at the pro-B stage [1], [3], [32]. These studies provide a foundation in support of the AMuLV-transformed pro-B cell model system for identifying miRNAs. They also support that miRNAs may regulate other transitions in B cell development such as the pre-B to immature-B transition that we observe.

Many miRNAs, including those expressed at the pro-to-pre-B transition, are described as being master regulators of key pathways. For example, miR150 (387168) was identified as a robust regulator at the pro-to-pre-B transition [2], [33]. B lineage-specific miR150 transgenic mice display a block at the pro-to-pre-B transition. Additionally, the miR17-92 polycistronic cluster knockout mouse has a block at the pro-to-pre-B transition in B cell development [3], also serving as an example of master regulatory miRNA potential in B cell development.

miR290-5p/292-5p knockdown, in either STI571-treated AMuLV-transformed pro-B cells or in an IL7 regulated primary B cell culture system, and germline-deletion of the miR290 cluster in mice, result in a blunting of κGT induction. Blunting rather than ablation of κGT induction indicates that these miRNAs contribute to, but are not essential for, this process. This is different than the robust effect of miR150 described above. This finding, together with the accumulation of the pre-B population in the miR290 cluster knockout mouse, underscores the potential of low-expressing miRNAs, such as miR290-5p/292-5p, that behave as modulators of pathways in developmental systems.

### miR290 Cluster and its Members

As described above, the miR290 cluster germline knockout has a partially penetrant embryonic lethality in which homozygotes survive gestation at only 7% of the predicted Mendelian ratio. Homozygous knockout mice that die in-utero have two major abnormal phenotypes. At E.10.5, 16% of homozygous knockouts develop outside of the yolk sac, while 40% of homozygotes have severe developmental defects including reduced somite number and delayed chorioallantoic attachment, among other things [7]. These defects are obvious contributors to the reduced survival potential of homozygotes, and emphasize the importance of the miR290 cluster in development. Our studies are the first to describe a phenotype in lymphoid cells in the miR290 cluster knockout mice.

In our study, miR290-5p/292-5p are thought to be similar in function since they share the seed sequence CUCAAA. This means that they are functionally redundant and target the same targets. The miR290 cluster members that share the seed sequence AAGUCC are expressed under different contexts. The differential expression of the CUCAAA and AAGUCC members in B cells may be attributed to differential maturation of the miRNAs in the miR290 cluster. Differential maturation may be due to pri-miRNA accessibility to Drosha, determined by the miR290 cluster tertiary structure, as is the case with the miR17∼92 cluster [34], among other possibilities.

Most recently, a role for the seed sequence-AAGUCC miRNAs but not seed sequence-CUCAAA miRNAs, was described in regulating DNA methyltransferases [35], the genes involved in apoptosis. In Dicer deletion studies of ESC [36], and in the regulation of the G1/S transition of the cell cycle in ESC. The AAGUCC miRNAs are characterized as proliferation-regulating miRNAs [37]. It is worth noting that in the ESC, the miR290 cluster AAGUCC members play the role of master regulators and strongly regulate proliferation. In our study we observe a more modulatory role for the CUCAAA members of this cluster, miR290-5p and miR292-5p. Interestingly, the AAGUCC miRNAs, including miR* counterparts miR290-3p and miR292-3p, are not detected in our system (Figure S1). There are few validated targets for miR290-5p or miR292-5p, but this group may include p16 [38] (12578) and Lats1 (16798), two genes implicated in cell cycling.

In summary, we identified two miRNAs, miR290-5p and miR292-5p, that are induced in pre-B cells. They increase binding activity of E2A and NF-κB to chromosomal Eκi sequences and modulate induction of κGT expression. κGT expression is critical to activation of the *kappa* locus for rearrangement of a functional immunoglobulin light chain. Our studies have uncovered a novel role for miR290-5p and miR292-5p in this key developmental process.

## Materials and Methods

### Ethics Statement

All mouse experimentation was approved by the Animal Care and Use Committee of the University of California at Berkeley (Protocol # R253-0313BR). The handling of the animals was in accordance with this protocol.

### Mice

xmiR290 cluster −/− mice, on a C57BL/6 and 129 mixed background, were a gift from the Sharp and Jaenisch Labs at the Koch Institute (MIT). C57BL/6 mice were purchased from Jackson Laboratories.

### Cell Culture and Retroviral Infections

Phoenix cells [24] were grown in Dulbecco’s modified Eagle’s medium (DMEM) supplemented with heat-inactivated 10% fetal calf serum, and sodium pyruvate (800 µM). AMuLV cell lines E2A+/+, 220-8 [25], and 63-12 [39] were cultured in RPMI supplemented with heat-inactivated 5% fetal calf serum, penicillin (100 µg/ml), 2 mM L-Glutamine, and 2-mercaptoethanol (50 µM).

MSCV-iRES-hCD2-miR30 was previously described [40]. The miR30 cassette was replaced with either miR129-2_3p, miR290-5p, or miR292-5p. The corresponding miRNAs* were mutated for each pre-miRNA. Primers used for miRNA cloning were as follows:

miR129-2_3p 5′ ACTGCTCGACCCCCTTCGAGCTACCCTTAT,

miR129-2_3P 3′ ACTGGCGGCCGCTCCCCCTGTGGTACAAAGTC;

miR290-5p 5′ ATCGGGATCCTCTGGGCGCGAGTGA,

miR290-5p 3′ ATCGGTCGACGCTCAAGAGAGGG;

miR292-5p 5′ ATCGGGATCCGTGGAGGCCCTCTCT,

miR292-5p 3′ ATCGGTCGACCCAAGCCGCGGAG.

Sponges were generated from long oligos with imperfect binding sites for miRNAs and were annealed and cloned into V26-MSCV-PIG. Long oligos used to generate each miRNA sponge contain three consecutive copies of binding sites for the corresponding miRNA with each binding site separated by a CCGG stuffer sequence. A bulge for imperfect binding was created corresponding to positions 9–12 of the miRNA. Binding sites were as follows: miR129-2_3p: ATGCTTTTTGTTTGAAGGGCTT, miR290-5p: AAAGTGCCCCACCCGTTTGAGT, miR292-5p: CAAAAGAGCCAAACGTTTGAGT.

Phoenix cells were transfected with above retroviral plasmids (12 µg) and a vector expressing vesicular stomatitis virus G (3 µg), using Lipofectamine 2000 (Invitrogen). Lipofectamine-DNA complexes were incubated at room temperature for 20 minutes, and added dropwise to Phoneix cells in Optimem (10% FCS), according to the manufacturer’s instructions (Invitrogen). Two days after transfection, viral supernatant was harvested and sterile-filtered (.45 µm filter). E2A+/+, HF4 AMuLV cells (2×10^5^) or primary bone marrow cells (six bones from one mouse) were transduced with 2 ml viral supernatant by spinfection (2500RPM, 2 Hr., 32C). After spinfection, volume was brought up to 4 ml with RPMI (complete) and cultured.

### ChIP

Chromatin Immunoprecipitation (ChIP) was conducted as previously described [26]. Recovered DNA was resuspended in 100 µl water. Primers used for PCR analysis of recovered DNA were as follows: Eκi F’TTAAGGCCTGTCCATACAGT; Eκi R’ATGTTTGGGAGTCTGAACAC; NF-κB F’ACCTCTGTCACCCAAGAGTTGGC; NF-κB R’ACAGGGCCTTAAGCCAGGGTC.

### qRT-PCR

Total RNA was isolated with Trizol Reagent (Invitrogen) and reverse transcription was performed with random hexamers and Moloney MLV reverse Transcriptase (MMLV) (Invitrogen). Quantitative reverse transcription PCR (qRT-PCR) was performed with Jumpstart *Taq* (Sigma), EvaGreen (Biotium), on an ABI 7300 Thermocycler (Applied Biosystems). PCR amplification was as follows: 95C 3 min, 95C 30 sec, 60C 30 sec, 72C 30 sec (data collection), for 40 cycles. Primers used in this study were previously described [40]. Primers used for κGT were as follows:

κGT 5′: GGACGTTCGGTGGAGGC.

κGT 3′: GGAAGATGGATACAGTTGGT.

miRNA reverse transcription was conducted as previously described [40], using 50 ng of total RNA and miRNA-specific reverse-transcription primer (Assay IDs 001184, 002590, 001055, ABI). miRNA qRT-PCR was conducted as previously described [40], using miRNA-specific Taqman primer sets included in miRNA-specific assays.

### Flow Cytometry and FACS Sorting Analysis

Total bone marrow was harvested from mice and red blood cells were depleted with EryLyse Buffer (0.14 M NH4Cl, 20 mM Hepes). Cells were stained with anti-CD19 MACS microbeads (Miltenyi Biotec) and were CD19+ enriched using MACS MS columns (Miltenyi Biotec). CD19+ cells were then stained using the following antibodies: B220-PeCy5 (clone RA3-6B2, BD), CD43-Pe (clone S7, BD), IgM-FITC (clone II/41, eBiosciences), IgD-Biotin (clone 11–26, eBiosciences), Streptavidin-PeCy7 (BD). Stained cells were resuspended in FACS buffer (5% BSA, 10 mM Hepes) for cell sorting.

## Supporting Information

Figure S1 **Expression of alternative miR290 cluster members.** qPCR analysis of miR291-5p, miR290-3p, and miR292-3p expression levels in RNA purified from primary wild-type pro-B (B220+, CD43+, IgM−) or pre-B (B220+, CD43−, IgM−) cells. Data was normalized to the expression of miR129-2_3p. Error bars represent range for replicate qPCR reactions. Data shows one representative experiment of at least three independent experiments.
(TIF)

Figure S2 **miRNAs engage with knockdown-sponge construct.** FACS analysis of sponge marker, GFP, expression upon STI571 (1 µM, 42 hr) induction of miRNAs. E2A+/+ AMuLV cells were stably transduced with tandem tomato marked sponge constructs for miR129-2_3p, mir290-5p, or miR292-5p. Cells were gated on the tomato positive population and were analyzed for GFP expression upon STI571 treatment. (Grey fill, untreated cell line; Black line, STI-treated cell line).
(TIF)

Figure S3 **Over-expression of miR290-5p or miR292-5p do not decrease E2A mRNA expression.** QPCR analysis of E2A+/+ AMuLV cells expressing either an empty vector control, miR290-5p, or miR292-5p for (A) E12 or (B) E47.
(TIF)

Figure S4 **miR290-5p or miR292-5p do not directly repress the ID2 3′UTR.** Luciferase assay of total cell lysates from HEK293 cells transiently transfected with either a wild-type ID2 3′UTR reporter or a mutant ID2 3′UTR reporter along with a scramble miRNA, mir290-5p or miR292-5p. Error bars represent range for biological replicate luciferase reactions. Data shown is of one experiment representative of at least three independent experiments.
(TIF)
