# Supplementary figures and images for: miR290-5p/292-5p Activate the Immunoglobulin kappa Locus in B Cell Development
Source: PLoS One. 2012 Aug 23;7(8):e43805. doi: 10.1371/journal.pone.0043805 (PMC3426528; doi:10.1371/journal.pone.0043805)

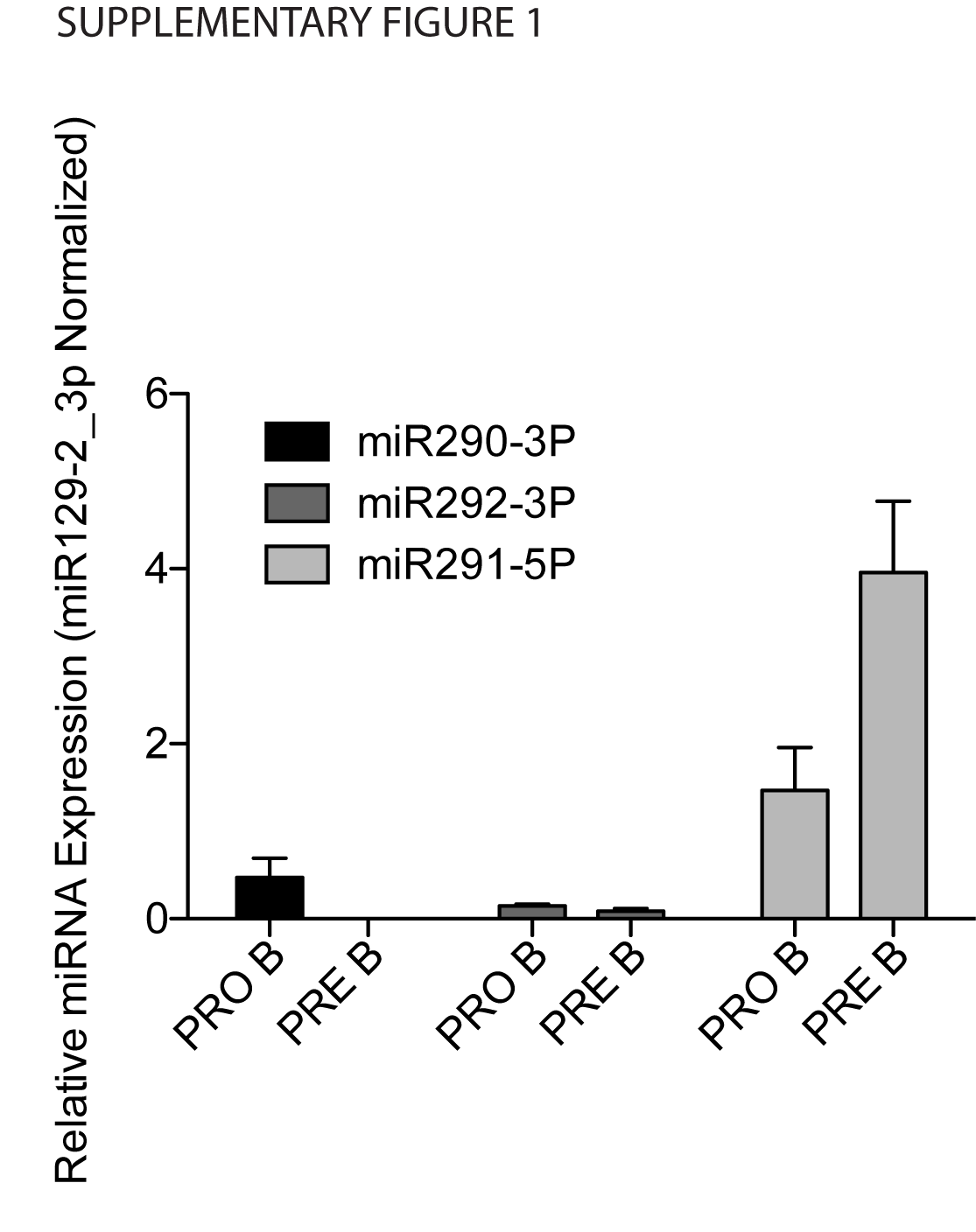

Supplement: Figure S1 — Expression of alternative miR290 cluster members. qPCR analysis of miR291-5p, miR290-3p, and miR292-3p expression levels in RNA purified from primary wild-type pro-B (B220+, CD43+, IgM−) or pre-B (B220+, CD43−, IgM−) cells. Data was normalized to the expression of miR129-2_3p. Error bars represent range for replicate qPCR reactions. Data shows one representative experiment of at least three independent experiments. (TIF) [file pone.0043805.s001.tif]

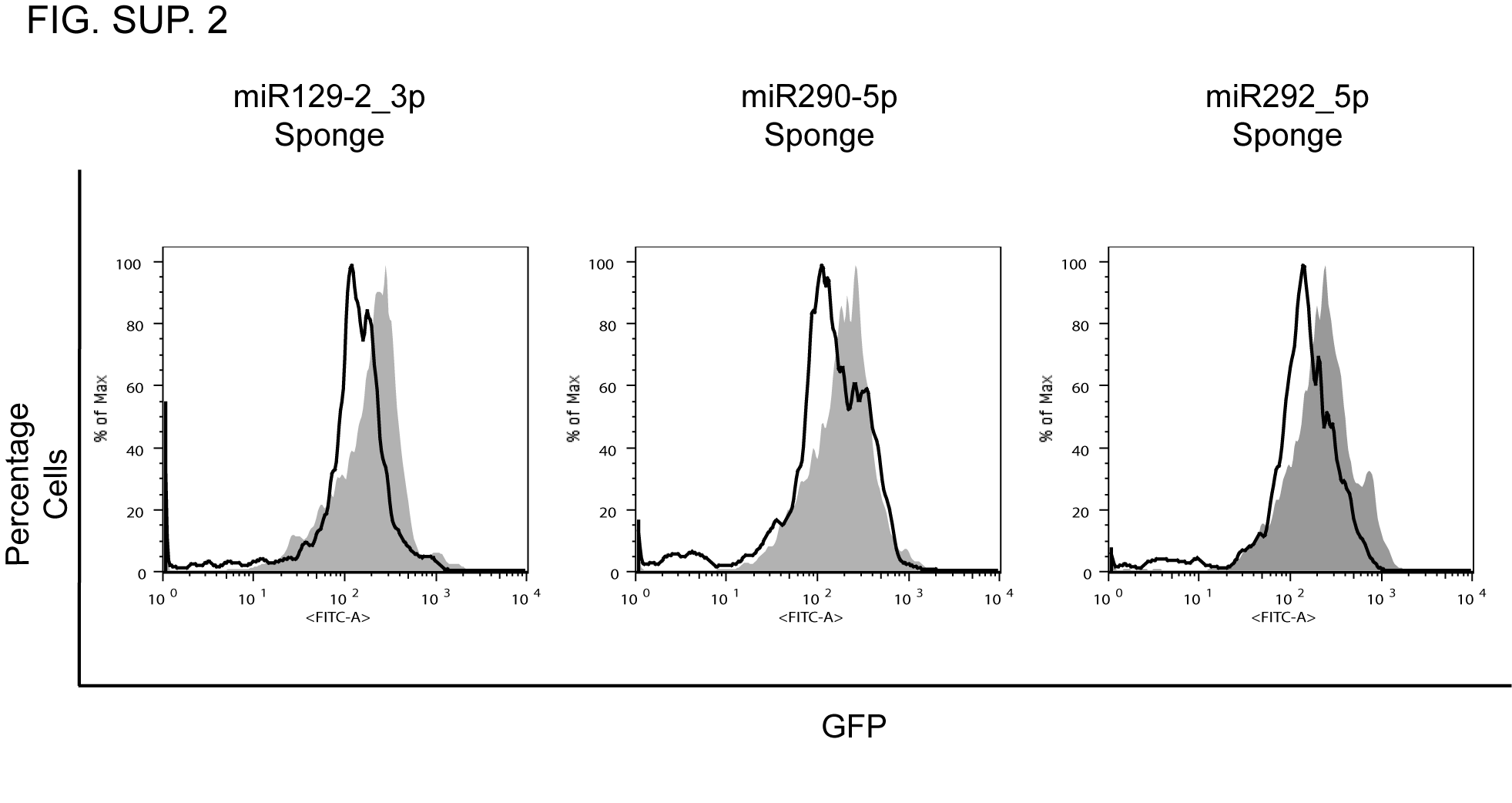

Supplement: Figure S2 — miRNAs engage with knockdown-sponge construct. FACS analysis of sponge marker, GFP, expression upon STI571 (1 µM, 42 hr) induction of miRNAs. E2A+/+ AMuLV cells were stably transduced with tandem tomato marked sponge constructs for miR129-2_3p, mir290-5p, or miR292-5p. Cells were gated on the tomato positive population and were analyzed for GFP expression upon STI571 treatment. (Grey fill, untreated cell line; Black line, STI-treated cell line). (TIF) [file pone.0043805.s002.tif]

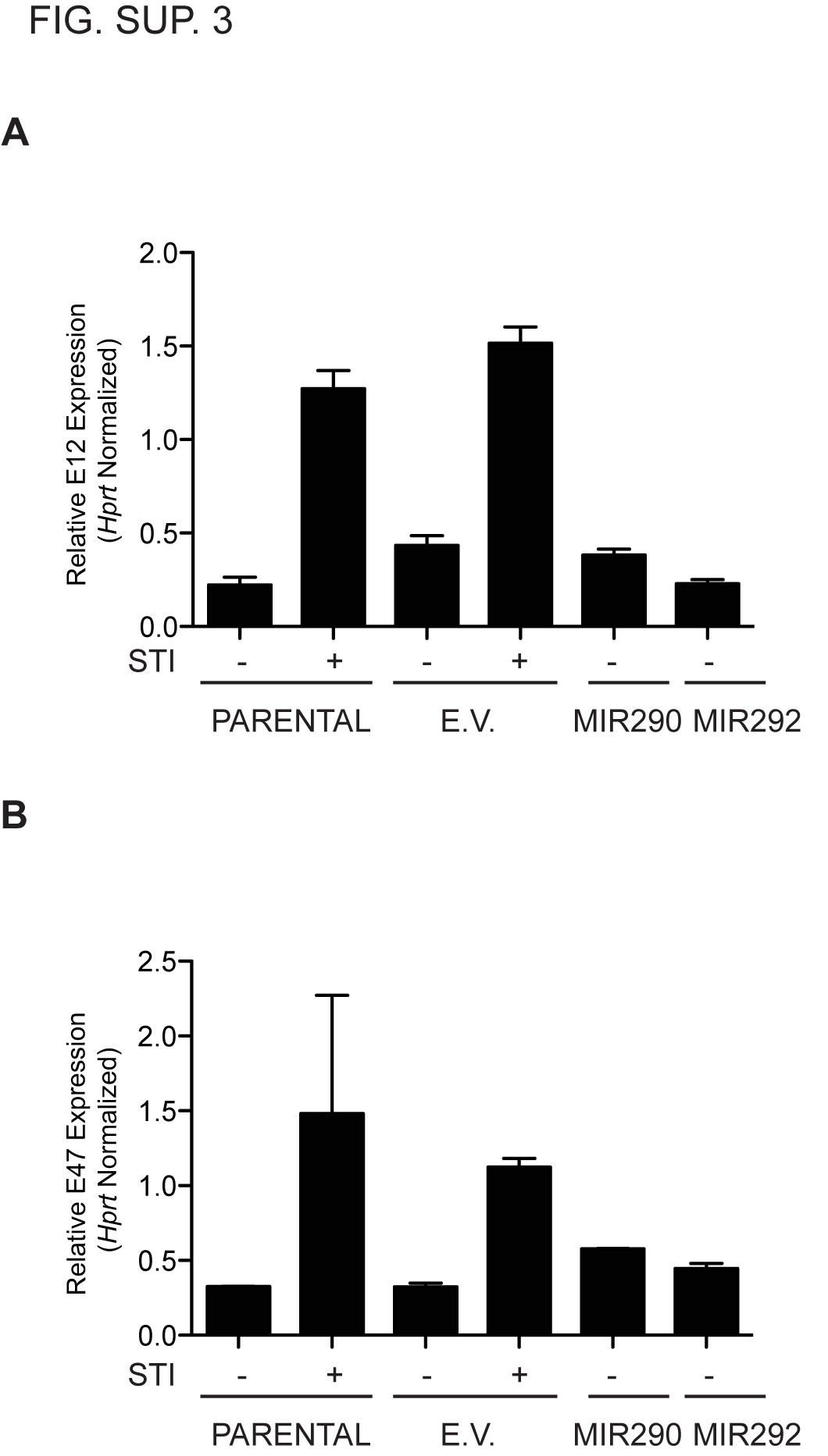

Supplement: Figure S3 — Over-expression of miR290-5p or miR292-5p do not decrease E2A mRNA expression. QPCR analysis of E2A+/+ AMuLV cells expressing either an empty vector control, miR290-5p, or miR292-5p for (A) E12 or (B) E47. (TIF) [file pone.0043805.s003.tif]

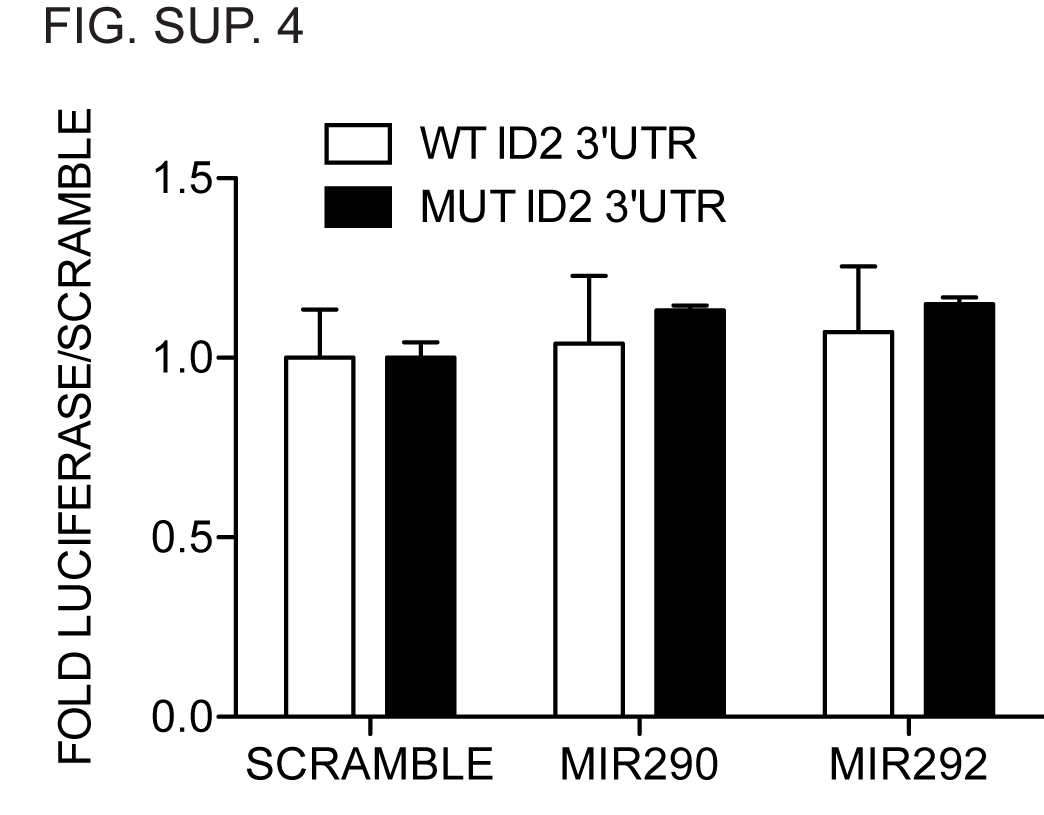

Supplement: Figure S4 — miR290-5p or miR292-5p do not directly repress the ID2 3′UTR. Luciferase assay of total cell lysates from HEK293 cells transiently transfected with either a wild-type ID2 3′UTR reporter or a mutant ID2 3′UTR reporter along with a scramble miRNA, mir290-5p or miR292-5p. Error bars represent range for biological replicate luciferase reactions. Data shown is of one experiment representative of at least three independent experiments. (TIF) [file pone.0043805.s004.tif]
